# “The Land Nurtures Our Spirit”: Understanding the Role of the Land in Labrador Innu Wellbeing

**DOI:** 10.3390/ijerph18105102

**Published:** 2021-05-12

**Authors:** Leonor Mercedes Ward, Mary Janet Hill, Nikashant Antane, Samia Chreim, Anita Olsen Harper, Samantha Wells

**Affiliations:** 1 Population Health Ph.D. Program, Faculty of Health Science, University of Ottawa, Ottawa, ON K1N 6N5, Canada; chreim@telfer.uottawa.ca; 2 Sheshatshiu Innu First Nation, Innu Nation of Labrador, Sheshatshiu, NL A0P 1M0, Canada; maryjanethill@gmail.com; 3 Innu Round Table, Innu Nation of Labrador, Sheshatshiu, NL A0P 1M0, Canada; aandrew@sifn.ca; 4 Telfer School of Management, University of Ottawa, Ottawa, ON K1N 6N5, Canada; 5 National Aboriginal Circle against Family Violence, Kahnawake, QC J0L 1B0, Canada; a_oharper@bell.net; 6 Centre for Addiction and Mental Health, Institute for Mental Health Policy Research, Toronto, ON M5S 2S1, Canada; Samantha.wells@camh.ca; 7 Centre for Addiction and Mental Health, Campbell Family Mental Health Research Institute, Toronto, ON M5T 1R8, Canada; 8 Dalla Lana School of Public Health, University of Toronto, Toronto, ON M5S 1A1, Canada; 9 Department of Psychiatry, University of Toronto, Toronto, ON M5S 1A1, Canada; 10 Department of Epidemiology and Biostatistics, Schulich School of Medicine and Dentistry, Western University, London, ON N6A 5C1, Canada; 11 School of Psychology, Deakin University, Burwood 3217, Australia

**Keywords:** Indigenous health and wellbeing, land, indigenous knowledge, cultural identity, self-determination in research, Labrador Innu, Canada

## Abstract

We examined Indigenous views of wellbeing, aiming to understand how the Labrador Innu view influence of land on their health. The Innu live in two First Nation communities (Sheshatshiu and Natuashish) in the subarctic portion of the province of Newfoundland and Labrador, Canada. Their views on land and wellbeing are context specific and have not been studied; our research addresses this significant gap in literature. Findings highlight that the experience of being on the land with family and community, learning cultural knowledge, and gaining a sense of identity play a major role in enhancing wellbeing. Externally imposed policies and programs conceiving Indigenous land as a physical place only fail to understand that land sustains wellbeing by emplacing knowledge systems and cultural identity.

## 1. Introduction

There are approximately 370 million Indigenous [1,2] peoples worldwide whose health status is significantly lower than that of their non-Indigenous counterparts [3,4,5]. Amongst these peoples, there is immense cultural diversity. In Canada, for example, Indigenous peoples are divided according to the Constitution into First Nations, Metis and Inuit [6], speaking over 70 languages within 12 distinct language families [7]. For Indigenous populations, given their cultural diversity, there is no one single understanding of contemporary Indigenous health and wellbeing [8,9], and health research with Indigenous peoples indicates that understandings of health and wellbeing are culture specific [10,11,12,13]. As such, improving the health of Indigenous peoples through health programming requires consideration of each community’s historical, geographical, and cultural contexts; without these considerations, the specific needs and concerns of the community are not met [14]. This paper examines views of wellbeing and how the land enhances wellbeing among the Labrador Innu, who reside in the province of Newfoundland and Labrador, Canada. This research will be used to inform the enhancement of community-based programming to improve the mental health of the Labrador Innu. 

The disparities in the health status of Indigenous peoples can be understood, at least in part, through a social determinants of health perspective, including a focus on income, education, employment, living conditions, and access to health services. Current Indigenous-specific disparities originate from historic and contemporary colonization, which result in continuing loss of culture, autonomy, land and health [15,16,17]. In Canada, the 1876 *Indian Act* legislation promulgated by the English Crown enacted policies of assimilation and further displaced the First Peoples from the land; then, the residential school system separated children from their families for the purpose of eradicating cultures, languages, and Indigenous identities. 

A salient aspect of these colonial practices in various countries around the world is the alienation of Indigenous people from their lands. Loss of land among Indigenous peoples has meant less time on the land and thus reduced opportunities for sharing knowledges on cultures, skills and practices, which, in turn, resulted in a weakening of social bonds and self-esteem associated with cultural identity [18,19,20,21,22,23]. The health implications of these losses are significant. 

There is great cultural diversity among Indigenous peoples and understandings of Indigenous health and wellbeing are specific to each culture [10,11,12,13]. One example of the specificity of cultural understandings of wellbeing is found in the Medicine Wheel. The Plains Cree of the Great Plains of North America encapsulate their concept of wellbeing in the symbol of the Medicine Wheel [9]. While the Labrador Innu and the Plains Cree speak Algonquian languages, the Medicine Wheel is not traditionally used by the Innu. Despite specificities, understandings of Indigenous health and wellbeing hold principles in common. One of the common principles has been noted by Indigenous philosophers of the Americas: all nature and all beings (humans and non-humans) have a purpose and a relationship to each other [24,25,26]. Similarly, Drahos [27], a non-Indigenous academic working with Indigenous peoples in Australia, upheld that Indigenous knowledges, land, and people exist in an integrated system in which humans and non-humans are all part of a “territorial cosmos”. The concept of territorial cosmos places Indigenous knowledges and people on a land inhabited by humans and non-humans. Indigenous geographers have also brought attention to the holism in the connections among Indigenous health, land, and cultural identity [13,22,28], highlighting as well the common recognition of land as a determinant to all or most Indigenous peoples’ health [21,22,29,30]. 

Nevertheless, in the same way that understandings of wellbeing are culture specific, interdependent relationships between Indigenous peoples and the land have also been found to be culture specific [10,11,12,13,31,32,33]. For example, Izquierdo [31] found that although the health outcomes of the Matsigenka people in Peru improved with more access to non-Indigenous health services, their sense of wellbeing simultaneously decreased. This is because the meanings and qualities they attribute to wellbeing include productivity (understood as providing for the family by being skilled hunters, fishermen and weavers), harmony (with their social, physical, and spiritual environment), and goodness (understood as the subordination of individual needs to the larger collective). For the Maori of New Zealand, wellbeing concepts include obligations towards their tribe, responsibility towards the land, and balance that requires maintaining relationships with, for example, the gods of the forests [21]. These examples show that there is diversity in perspectives of wellbeing.

The Labrador Innu (Innu hereafter) live in the communities of Sheshatshiu (Sheshatshiu Innu First Nation—SIFN) and Natuashish (Mushuau Innu First Nation—MIFN). The Innu have an intrinsic understanding of wellbeing although there is a lack of research and literature on their perspectives of the significant relationship between land and wellbeing. Our article helps fill this gap. This is essential research to better understand these inter-relationships which are specific to the unique context of a community, and also to inform Innu health policy and practice, especially when enacted by non-Innu. *Tshenut* (*Tshenut* is the plural of *Tshenu*, meaning old persons in Innu-aimun (the language of the Innu), and we use this word instead of Elder, capitalizing it to indicate respect.) know that the land is key to wellbeing [34]. In a community-initiated larger study supported by the then Grand Chief of the political umbrella organization of SIFN and MIFN, Innu Nation, the Innu partnered with outside researchers on a larger study on Innu mental health and wellbeing, involving researchers from both MIFN and SIFN. This article reports on Innu views of wellbeing (*minuinniuin*), including how the land contributes to their wellbeing. Grand Chief Anastasia Qupee specified that this research be conducted fully utilizing Innu ways of knowing and knowledge systems. All subsequent Grand Chiefs have been supportive of this community-initiated research. Through a community-based participatory approach, we answered the following research questions: How do Innu view wellbeing? How does the land support wellbeing?

For this article, the authors are Innu and non-Innu researchers as follows: two Innu (second and third authors); one Indigenous non-Innu scholar (fifth author); one mixed (non-Innu) South American Indigenous scholar (first author—L.W.), who lived in Labrador and worked for the Innu; two non-Indigenous scholars (fourth and sixth authors). All authors share a commitment to understanding and improving Indigenous health and wellbeing, and a perspective that Indigenous knowledges contain important understandings of the world.

The first section of this article briefly presents Innu history relative to their connection to the land, and notes the events that displaced them from their ancestral territory—as well as their response of cultural revitalization. This history has impacted their health and wellbeing. The second section outlines the methods used in this study. Next, the research results are presented, followed by the findings and implications.

### 1.1. Overview: Innu Traditional Life, the Land, Health Status and Current Policies

*Nutshimit* (*Nutshimit* is an Innu-aimun word translated into English as land, country, outpost. We write *Nutshimit* in capital letters to indicate in written language the respect Innu have for the land.) has been the place of totality in Innu life for millennia. Until the 1950s, the Innu were self-sufficient nomadic hunters in the subarctic environment of the Quebec–Labrador Peninsula. They lived as interdependent people who traversed vast lands in a 800,000 km^2^ territory called *Nitassinan* (meaning “our homeland”) [35]. Today, they are settled in eleven communities—nine in the province of Quebec, and two in the province of Newfoundland and Labrador (NL), Canada. This study pertains to Natuashish and Sheshatshiu in NL. Collectively, they have a population of approximately 2200 [7]. 

#### 1.1.1. Traditional Innu Life

Survival on the land requires exactitude and cooperation, with everyone contributing their skills and labour. Children learned what was required to survive on the land by approximately 15 or 16 years of age; they were taught by parents and grandparents everything from hunting, cooking, erecting tents, planning hunting expeditions, raising children, finding medicine, and interpreting the weather. Until the 1950s, the Innu travelled for 9 months of the year [36] in groups of approximately 3 to 5 couples with their unmarried children. The small groups assembled briefly in larger gatherings at places where resources were plentiful. In these gatherings, they traded and made marriage alliances [37].

Life on the land saw the Innu in permanent relationship with all living beings (human and non-human). One example is the traditional hunting philosophy of *respecting the animals* [37,38]. The Innu word for caribou is *atiku*, a non-human living creature with full personhood and will, and inhabited by spirit. Hunting, then, is not about outsmarting animals but about enticing fully volitional beings to be generous with their bodies [39]. The peoples’ respect is such that *atiku* return each year to provide for Innu needs (i.e., meat and marrow for food, bones for spiritual ceremony). This relationship supports the claim of Indigenous philosophers that although there is great diversity of cultures and understandings of health and wellbeing, there are common principles that all living entities exist in relationship to each other [24,25,26].

The Innu continue hunting today, mostly in spring and autumn. Contemporary hunting is described as requiring tremendous amounts of energy, coordination between mind and body, alertness, quick decision-making, improvising in the subarctic world, and an understanding of the constant changes in the weather and its effects on the movements of animals [40].

#### 1.1.2. Policies and Their Effects on Innu Health and Wellbeing

Policies of forced settlement brought an abrupt end to traditional life [41], effectively dispossessing the Innu from their land. Innu hunters provided for their families and the larger Innu community by maintaining kinship bonds with the land that were renewed as they walked the land while they hunted. Henriksen [42] documented stories of Mushuau hunters in the 1970s where these bonds are manifested in mutual care for each other (Innu and land), allowing hunters to survive in the barren lands of northern Labrador. This understanding of relational bond with the land is shared among many other Indigenous peoples and expressed in their creation stories where people come into being when the land comes into being [43]. Such understanding of land is different than European concepts of land that influenced the English Crown’s promulgation of the Indian Act that declared Indigenous land in Canada as “property” of the Crown. The European view of land at the time of the promulgation of the Indian Act had its origins in the scientific revolution [44]. The scientific revolution developed an epistemology based on the distinction between nature and culture. Within this paradigm, man is defined by the attainment of culture through civilization, and nature is defined by lack of cultural qualities. Thus, in this paradigm, man’s relationship to nature becomes one of mastery, subjugation and taming through notions of ‘improvement’ and ‘progress’, and Indigenous peoples are conceived in the category of nature [44].

Until the mid-twentieth century, the federal and provincial governments in Canada upheld concepts of ‘improvement’ and ‘progress’ that considered Innu life on the land as uncivilized [45] and required government intervention through forced settlement and schooling. Mushuau Innu who now live in Natuashish were forcibly relocated in 1948 to the coastal community of Nutak. After one year, they walked back to traditional territory and returned to the hunting life. The second relocation was to Davis Inlet in 1967 [45]. They were given poorly insulated homes with plumbing infrastructure but no running water. In 2002, they were relocated to Natuashish, a place of their choice. The Sheshatshiu Innu were settled in the 1960s in the location that they gathered at for centuries, close to a trading post at North West River.

Through provincial government policy, traditional Innu hunting became illegal and children’s enrollment in non-Innu schools was mandatory [41,46]. Forced schooling had a detrimental impact for the preservation, practice, and intergenerational transmission of knowledge which takes place on the land. Making hunting illegal changed the role of men as providers for their families, and as teachers of younger men; this caused dependency on government welfare for families’ sustenance. Forced schooling also affected *Tshenut* who have a societal role as knowledge holders [47], cultural transmitters [48,49], and safeguarders of the worldview [50]. 

Gregoire [46], an Innu man who experienced land dispossession, described policy changes as initiating a pattern of alcohol abuse, violence, family breakdown, suicide, accidents, and illness. His view is shared by *Tshenut*, who identify land dispossession as the point when social ills began [45,51,52]. Land dispossession was furthered through mining development, flooding of lands for a hydroelectric project, and an increase in NATO military activity over Innu traditional territories. 

#### 1.1.3. The Emergence of Cultural Revitalization

Despite government efforts, the Innu never stopped finding ways of returning to the land. It was their worldview that mobilized them to engage with the federal government for the first time in 1980. As noted by Mailhot [37], Innu truth is that one takes care of the land and land takes care of people. In 1979, NATO low-level fly training over hunting camps became too disturbing to the families and to the animals. Military planes from European allied nations were based at the airport in Goose Bay (40 km from Sheshatshiu) and conducted training at low altitudes over lands considered ‘unpopulated’. Innu wrote to federal ministers in protest for a period of six years, and garnered support of NGOs and the international media [53]. In the late 1980s, in a tremendous showcase of strength to defend their lands, Innu engaged in contentious collective action by occupying the runways of the military airport in Goose Bay on multiple occasions. As a result of the protests, the government extended the Indian Act in 2002 (a century-and-a-half later than most other First Nations in Canada), and agreed to a settlement of land claims after noticing that public opinion was favourable to the Innu [45]. Land claims were signed in principle in 2011, but to date, have not been finalized. 

Another successful Innu negotiation was funding for families to go back to the land. Known as “outpost”, these finances covered the cost of families for hunting in the spring and fall, and to revitalize their culture. The funding, though, has remained unchanged in its nominal amounts over the years (personal communication with Band Councils, October 2015). 

The protests confirmed that the Innu are a people with knowledge and understanding of their lands [40] and generated many activists. One is *Tshenu* Taukuesh Penashue, who leads younger Innu on traditional walks in a rediscovery of their culture. Traditionalists such as Taukuesh engage in activities that help younger Innu repossess a way of life on the land. 

When the Innu came under Indian Act authority, new Western-based health practices arrived in their communities. However, these failed to acknowledge how land dispossession has negatively affected the wellbeing of the people [45]. Innu responded by advancing their self-determination through progressive steps, one of which was creating the Innu Round Table (IRT) in 2012, as the executive arm of Innu Nation. After extensive community consultation the IRT articulated a Healing Strategy [34]. The Strategy conceives a contemporary return to land activities as the foundation for health and wellbeing for the rebuilding of their communities. Innu self-determination has many fronts including doing research initiated by Innu in areas of interest to them, such as the current project which aims to better understand Innu wellbeing and its connection to the land. 

## 2. Methodological Approach

### 2.1. Analytic Framework and Ethics

Our approach was community-based participatory research (CBPR) [54] using an Innu framework for health research developed at the start of the research partnership [55] from a larger study on mental health and wellbeing that is ongoing in the two Innu communities. This research aimed to engage all partners dialogically in an ethical space [56] where relationships among all researchers would utilize Innu knowledge and ways of knowing [55]. The team, including Innu and non-Innu researchers, was guided by OCAP^R^, a registered trademark of the First Nations Information Governance Centre [57], that protects self-determination in research through collective ownership, control, access and *p*ossession of information. A Core Research Team (CRT) under the advice of *Tshenut* was formed to steer the research led by the Innu. Ethics approval was obtained from the Innu Nation, the NL Research Ethics Board, and two academic institutions in Ontario, where LW and the Principal Investigator work.

### 2.2. Data Sources and Collection

Participants 16 years of age and older who were living in the communities and self-identified as Innu were recruited by Innu co-researchers and the Community Youth Coordinators (CYC) of each of the communities. CYC are adults employed by SIFN and MIFN. Data collection was conducted under the oversight of the CRT from January to June 2019. Data collection involved individual semi-structured interviews and focus group discussions to explore Innu views of wellbeing, and how land enhances wellbeing. Guiding questions for the interviews and focus groups were translated into Innu-aimun, reviewed and approved by the CRT and the CYC. Participants were asked, for example: “what does *minuinniuin* mean to you?”, “what/who makes it possible for you to feel well?”, “can you describe what you feel in *Nutshimit*?” Recruitment continued until there were no new themes in new interviews and focus group discussions, indicating that we had reached data saturation. Twenty three participants were interviewed and 16 participated in focus groups, for a total of 39 participants from the two communities. During this study, LW was invited by *Tshenut* and leaders to participate in community gatherings on the land and the sacred feast of the *mokoshan* (The feast of the *mokoshan* is a communal spiritual celebration where Innu partake in a meal prepared with the bones and marrow from the legs of the caribou.) during the fall of 2018 and winter of 2019. These events offered contextual information for this study.

LW conducted the individual face-to-face interviews in English at a local community centre. These interviews lasted between 35 and 75 min each. Focus groups were also facilitated by LW in English, with the participation of youth between 16 and 19 years; these were hosted by the CYCs at the youth centre of each community. Each focus group lasted approximately 2 h, and at the end of each, participants celebrated with a shared meal. Interpreters were available at all focus groups and interviews, as required by participants. Written informed consent was obtained from all participants, who were offered a $30 gift certificate prior to their participation. *Tshenut* who participated in research meetings were given honorariums to indicate respect and thankfulness for their stories. All interviews and focus groups were audio-recorded and LW transcribed them verbatim. To protect privacy and confidentiality, Innu co-researchers assigned Innu names to all participants.

CBPR involves a process of reflection and articulation of experiences and perceptions on the part of non-Indigenous researchers or Indigenous researchers who are not members of the community [58,59]. An important limitation of our study was that non-Innu researchers do not speak Innu-aimun and the interviews and focus groups were conducted in English and translated back by interpreters. The Labrador Innu have maintained their language at incredible high rates [7] (close to 90%) and English is their second language. We mitigated the effect of multiple translations by maintaining a close dialogue in all research stages among all the researchers, Innu and non-Innu.

### 2.3. Analysis

The team conducted a thematic analysis [60] in an iterative manner following several steps. First, the transcribed interviews and focus groups were preliminarily explored by becoming familiarized through several readings and writing of memos (LW). Next, LW analyzed and generated initial themes (e.g., “family and community”, “Innu knowledge”) expressed explicitly in relation to the research questions. Initial themes were discussed and adjusted by all co-researchers through several iterations on several occasions between June 2019 and November 2020. The final themes were applied to the entire data set. Findings were shared with the CRT at several stages of the analysis, allowing for guidance on the interpretation and organization of the results. In the following, we provide extensive quotations to illustrate and support the themes in the Findings. It should be noted that the various themes are not mutually exclusive; rather they are inter-related and the quotations we provide illustrate these inter-relationships. 

## 3. Findings

In this section, we report on Innu views of wellbeing as well as the role of the land in enhancing *minuinniuin*, which the Innu translate into English as “wellbeing”. We organized the findings around the themes that emerged from the analysis.

### 3.1. Minuinniuin (Wellbeing) as Life on the Land

In response to the question, “what does *minuinniuin* mean to you?”, participants noted that it is subjective (“*something you feel*”, *Tshenu* Pinip, age 60s) and that it is the aim or destination of a process that occurs throughout one’s life *(“We keep going to minuinniuin, we keep traveling there”*—Napeu, age 19). *Minuinniuin* was depicted as “beautiful” (“*Minuinniuin is a beautiful thing.*” Puna, age 50s). Most described it as life in *Nutshimit* (the land, the country), where there is integrity and self-reliance—including the provision of food and medicine. In traditional Innu life, food and medicine come from the land, and people are self-sufficient:

*“Minuinniuin is life in Nutshimit. It helps our health because we eat from the land.” *

*(Puna, age 50s)*

*“I have minuinniuin on the land.” *

*(Nashtish, age 50s)*

*“Good food, traditional food, that is minuinniuin … when the mother eats the food that the husband hunts … the baby grows because the husband hunts the food the mother eats, and the baby eats what the mother eats.” *

*(Tshenu Shushtin, age 80)*

*“Minuinniuin is living a simple life... a humble life, being able to feed your children, to have what you need in your tent, and to be happy. If you were sick, you would go outside to find your medicine outdoors and make yourself better.” *

*(Tshenu Nishapet, age 70s)*

### 3.2. Land as a Place of Freedom

For most participants, land contributes to wellbeing by facilitating “freedom to be Innu”, away from the *akenashaut* (outsiders) gaze and expectations:

*“In Nutshimit we are free to be Innu; we are free to be who we are.” *

*(Maniakat, age 40s)*

*“Akenashaut cannot tell us what we can do and what we cannot do in that world.” *

*(Mani Shanet, age 27)*

*“[In Nutshimit] I feel free because everything around you is nature, water, nothing is going to stop you, no one is going to stop you, nobody is going to tell you what to do. It is all free, the water is free.” *

*(Shushep, age 24)*

Contrasting their feelings about the community where they experience a lack of autonomy and control, and worry about food security, some participants described how they felt about being on the land as a source and place of wellbeing:

*“[In the settled community] you are worried all the time about how you are going to pay your next bill and feed your kids, you are dependent on the store-bought food, on cash, and on your job so that you can pay your bills.” *

*(Nikashant, age 50s)*

*“As an Innu, I should be in the wilderness, but I am at home all day … There is too much electronics, I look at my phone all day, I look at my computer all day, I look at my TV all day, and I go to sleep. Get up in the morning and the same thing again.” *

*(Shimiu, age 32)*

### 3.3. Land as a Place of Togetherness and Relationship with All Living Beings 

In *Nutshimit,* families live together and this togetherness is part of *minuinniuin.* On the land, Innu individuals are part of a community that shares resources and also sorrows, providing a sense of belonging:

*“[Minuinniuin is when] everybody helps out and that brings the closeness in people. That is the way people have always been in Nutshimit. They always helped each other, they shared their food and their game.” *

*(Nashtish, age 50s)*

*“[Minuinniuin] is what you feel when something happens, and people gather around you and the family that is suffering. These relationships to family are very positive.” *

*(Mani Shanet, age 27)*

In *Nutshimit* the Innu live in a close relationship with the land and the spirits of the animals; these relationships were described as part of *minuinniuin*. The land, water and trees are living beings that the Innu strive to live in harmonious relationships with, as they connect with all living beings in a larger cycle of life renewal. These connections are facilitated by being on the land, and also through spiritual practices. Through drumming, dreaming, praying Innu have reciprocal relationship with spiritual forces:

*“The land nurtures our spirit.” *

*(Inushkueu, age 40s)*

*“[Minuinniuin] is remembering that everything is alive, even the snow is living, and that the trees are growing and everything grows back in the summer time” *

*(Puna, age 50s)*

*“Minuinniuin is being on the ground [on the land], it is to stay connected with the animals. Drumming connects you to the animal spirits, the drum can give you a message... dreams come when there is a message” *

*(Manian, age 40s)*

Some spiritual practices are performed as a community, such as the feast of the *mokoshan* to commune with and thank the caribou spirit for their provisions. This feast is a meal prepared by the hunters and the leaders with a special regard to caribou bone marrow, a sacred time that positively affects the wellbeing of Innu people:

*“What helps me to have minuinniuin is the mokoshan. It is the food we are eating [in the mokoshan] and also the people who come. My blood sugars are normal for the whole day and the day after” *

*(Shimu, age 32)*.

### 3.4. Land as a Place to Learn Innu Knowledge and Identity and Key Role of Tshenut in Teachings

Innu knowledge was described as part of *minuinniuin*, which is specific to their culture, and traditionally learned on the land. An example of this is recognizing thin ice (“*Our language has many words for snow or ice. You need to know the snow and the ice, or you will go through ice on a lake.*”—Shapatesh, 24). Innu knowledge is learned by observing, experiencing and engaging in cultural practices, listening to *Tshenut*, and also through introspection. A child learns by observation, then doing what he/she observes members of the family and *Tshenut* do (“*The boys learn to hunt and the girls learn to make bread. My wife taught the girls and I taught the boys*”—*Tshenu* Nuk, age 60s). 

Listening to and interacting with *Tshenut* is a cultural practice to access the wisdom of past generations, and is referred to as “*going to a living library*” (Shimun, age 50s). This contributes significantly to Innu wellbeing. Some *Tshenut* acquire deep spiritual understanding and work at passing this to the younger generations:

*“I listen to the Tshenut when they are talking about their ways in the past. Hearing them is really minuinniuin. There are times when I am all over the place and I have no patience. Tshenut help me see things the right way, [they help me] to calm my mind and to have patience … There has to be patience even in your dreams, and you have to keep praying … and you have to remember that everything grows back in summer time.” *

*(Shunin, age 50s)*

Shunin’s quote reveals that *Tshenut* teach a way of thinking, of look at life and seeing through their traditional worldview. Moreover, the teachings include hopefulness for the future (e.g., “*you have to remember that everything grows back in summer time*”). 

Participants also internalized *minuinniuin* as cultural understanding and discovering their own strength, and using this strength to form a positive Innu identity (a self-understanding about being Innu): (*“On the land you need knowledge to survive. When young people experience how hard it was for the Innu to survive they are very proud. They learn that they can do anything! We Innu are strong… this is minuinniuin.”—*Mani Shunin, age 50s). Thus, a positive Innu identity, also a part of *minuinniuin*, is associated with Innu knowledge. 

For *Tshenut*, a positive Innu identity is core to wellbeing (*minuinniuin*): “Knowing who you are is good medicine”—*Tshenu* Katnen, age 70s; “*minuinniuin* means knowing your identity, it is about being strong, not fearing”—*Tshenu* Pinip, age 60s. Positive identity is communicated by *Tshenut*, often on the land, by relating *Nutshimit* as the place of ancestors.

*“I went to Nutshimit [for the first time] when I was about 20 years old. I felt ashamed of who I was… not knowing what is to be Innu … I struggled … but one day we were gathered in a tent and a Tshenu presented the Innu timeline. I felt I was getting my identity back by hearing what the Innu went through. Even if I do not know a lot [of traditional knowledge], I still experienced that feeling of knowing who I was as an Innu person … once I tapped into seeing, feeling, being Innu, that is what helps me. Now I am proud of being an Innu person. That shame feeling is gone. [Now] I always keep in mind that the Innu people are strong, so many changes that happened in such a short period of time.” *

*(Enen, age 30s)*

### 3.5. Inter-Relationships among the Elements of Minuinniuin

The various elements of *minuinniuin*—land as place of freedom, togetherness and relationship with all living beings, Innu knowledge and identity—are inter-related and enhanced by each other. The following story of Tanien (age 17), who had been taken to *Nutshimit* by his grandfather since he was a child, discloses how culture is learned by observing and doing and by understanding the spiritual practices of Innu culture and knowledge. These link to strong identity and wellbeing, and foster a sense of freedom:

*My grandfather took me hunting … I remember that we got an animal and he took the antlers and put them on a tree. He said, “I am showing things that this land has given me; it gave me food and supplies.” We hung [the antlers] up and he was speaking in Innu and he said, “Thank you for providing for me.” He thanked the ancestors because they were here a lot longer and took care of everything [in the land]... I said, ah... respect, I want to say [respect] to the land in general, but it is more than that (long silence). An Innu person is giving, grateful, respectful, loving. They are people that can give out a hand, that can provide. An Innu man is one of the most respectful and respected, they work hard, they do everything they can to provide not only for their families but to others. I realized there what it meant to be Innu … is that traditions that are taught to me are entrusted to me so that I can pass them on too... Whenever I practice traditions I feel free, I feel like I can fly, it is an amazing feeling. *

*(Tanien, age 17)*

In Tanien’s story, he recalls the experience of being on the land and how it led to introspection and realization of his identity and purpose. This came from understanding why his grandfather was respectful to the animals, and thanked the land and his ancestors—an integral part of Innu spirituality. Moreover, Tanien connects the spiritual practice of respect as one that expresses how an Innu person is or should be (“giving, grateful, respectful”). He then affirms that practicing his culture, which he is entrusted to learn and pass to the next generations, gives him wellbeing (“I feel free… I can fly… amazing”), and confirms his identity. 

Participants indicated that *minuinniuin* also refers to wholeness beyond the physical dimension (“It is more than just the body, it is also the emotions and your spirit.”—Uapukun, late 30s; “[*minuinniuin* involves] the four areas like mental, spiritual, emotional, and physical, all those areas; you are whole then.”—Puna, age 50s). 

There is congruency in the understanding of what *minuinniuin* means for Innu people across the generations, independently of proficiency of culture on the land. This congruency can be described in the notion of *“Innu heaven”* brought up by participants. Innu heaven has all the elements of traditional life, with ancestors in the same ‘place’ (“*Innu heaven has a big tent where everybody is together with the ancestors … [The tent] is by the water in a beach, and you get there in a canoe; all the time there is a canoe, that is how you get to places.”*—Mani Shanet, age 27). 

Overall, Innu view *minuinniuin* as a subjective and aspirational state of being that is intricately related to *Nutshimit,* a ‘place of wellbeing’ that can be translated as land. For Innu, land is core to wellbeing. Land provides and facilitates togetherness, relationship to all living beings (humans and non-humans), enactment of Innu culture through which Innu worldview is maintained and taught, and finding of Innu identity.

## 4. Discussion and Conclusions

Our study is community initiated and leader supported. This study focusses on what is concerning to the two Labrador Innu communities of Sheshatshiu and Natuashish: the articulation for non-Innu audiences of their concept of wellbeing (*minuinniuin*), a needed first step for Innu-created health programming. We described the Innu concept of *minuinniuin* and showed that *Nutshimit* (land) is at its core. *Nutshimit* is a place where Innu live in permanent relational exchange with living humans and non-humans. While Innu experienced an abrupt change in their traditional way of life starting in the mid-twentieth century, they maintained their culture and their connection to the land.

Our findings highlight Innu views of wellbeing, including non-physical influences from experiences on *Nutshimit* where there are emotional, mental and spiritual benefits from being together as family and community. *Nutshimit,* the place where Innu learn cultural knowledge, provides them an identity as strong and free people, and autonomy through learning how to survive in this environment. Our findings illustrate the specificity of the relationships among *Nutshimit,* knowledge, identity and wellbeing, which is consistent with findings from research conducted by Indigenous geographers and allies [13,22,28]. Our research adds to the body of literature that recognizes land as a determinant of Indigenous peoples’ health [21,22,29,30]. 

We highlight the role that *Tshenut*, and Indigenous Elders in general, have in teaching knowledge experientially on the land, and how this affects wellbeing [48,49]. The continuous practice of culture brings Innu to a realization of dependence on each other, the weather, and the animals, in what becomes a spirituality and a worldview [49,50,61]. Our findings demonstrate that *Nutshimit* represents a place of freedom away from colonizing structures that are part of settled community life. This finding resonates with recent work on the health of Inuit of Labrador [19], which also reported experience of freedom when on the land. We also propose that the freedom Innu experience on *Nutshimit* is one of ontological wellbeing—a mode of “being-in-the-world” [50,61]. For the Innu, “being-in-*Nutshimit*” is “being at home”.

Our study contributes to knowledge of Innu contemporary understandings of wellbeing and the relationships between land (*Nutshimit*) and the people. While there might be other views of wellbeing, we only articulated the most commonly-reported perspectives by the participants. Our research provides outsiders (non-Indigenous) with understandings that can better shape Indigenous health programming in Canada’s North [62], where Innu live. Policy makers and health planners must be able to conceive of *Nutshimit* as more than land as physical space, and that *Nutshimit* sustains Innu *minuinniuin*. Without this understanding, we will continue to see health and wellness programming and service provision that are not culturally safe, perpetuating modern-day colonialism.

Moreover, these findings provide a foundation for reinforcing support for Innu land-based wellness programming (e.g., facilitating families going to the land, offering land-based experience to children at school, producing land-based curriculum), and the contemporary role that *Tshenut* are taking in passing on their teachings to the younger generations (e.g., inviting *Tshenut* to teach cultural activities as part of the school curriculum). While land-based activities have not been conceived as ‘programs’, we suggest that they be viewed as such given the importance of *Nutshimit* as core to *minuinniuin* (wellbeing). We suggest that health programming be developed with consideration of the importance of the land in improving a sense of wellbeing among the Innu peoples, as this would indeed bring Innu bodies, minds, emotions, and spirits “home”.

## Data Availability

Not applicable.
